# EOGD: the *Euplotes octocarinatus* genome database

**DOI:** 10.1186/s12864-018-4445-z

**Published:** 2018-01-19

**Authors:** Ruan-lin Wang, Wei Miao, Wei Wang, Jie Xiong, Ai-hua Liang

**Affiliations:** 1 Key Laboratory of Chemical Biology and Molecular Engineering of Ministry of Education, Institute of Biotechnology, Shanxi University, Taiyuan, 030006 China; 2 Key Laboratory of Aquatic Biodiversity and Conservation, Institute of Hydrobiology, Chinese Academy of Sciences, Wuhan, 430072 China

**Keywords:** *Euplotes octocarinatus*, Genome, Database

## Abstract

**Background:**

*Euplotes*, a ciliated protozoan, is a useful unicellular model organism. Studies on *Euplotes* have provided excellent insights into various basic biological principles. We have recently sequenced the macronuclear genome of the common freshwater species *Euplotes octocarinatus* to provide novel insights into *Euplotes* genetics and molecular biology.

**Results:**

In this study, we present the *E. octocarinatus* Genome Database (EOGD), a functional annotation and analysis platform for the global study of the *Euplotes* genome. EOGD includes macronuclear genomic and transcriptomic data, predicted gene models, coding sequences, protein sequences, and functional annotations. The GBrowser and BLAST tools are embedded in EOGD to enable the search, visualization and analysis of *E. octocarinatus* genomic and transcriptomic data.

**Conclusions:**

EOGD is a useful resource for the research community, particularly for researchers who conduct genome-scale analysis and molecular biology studies of *Euplotes* or other ciliates. EOGD will be continuously updated to integrate more datasets and analytical tools. EOGD is freely available at http://ciliates.ihb.ac.cn/database/home/#eo.

## Background

*Euplotes* is a free-living unicellular eukaryote that belongs to the ciliate phylum. This phylum also includes the model organisms *Tetrahymena, Paramecium* and *Oxytricha.* Like most ciliates, *Euplotes* exhibits nuclear dimorphism, possessing a germline micronucleus (MIC) and a somatic macronucleus (MAC). MIC is diploid, transcriptionally silent, and enables the transmission of genetic information between generations by sexual reproduction. MAC is generated from post-conjugation MIC, which is transcriptionally active during vegetative growth. Similar to other spirotrichous ciliates, *Euplotes*’s macronuclear chromosomes are tiny (nanochromosome) and mostly encode single genes that are differentially amplified to thousands of copies each [1, 2]. Two fundamental differences distinguish *Euplotes* from other ciliates: (i) the UGA codon of *Euplotes* is reassigned as cysteine or selenocysteine [3, 4]; and (ii) programmed ribosomal frameshifting (PRF) is widespread in *Euplotes* [5, 6].

Given its easy collection and laboratory cultivation, *Euplotes* is an attractive experimental system for studying basic eukaryotic biological processes. This organism has contributed to the discovery of fundamental eukaryotic mechanisms, such as the key telomerase protein [7], genome reorganization [8], defensive changes in cellular architecture in response to predator-produced signals [9], pheromone signaling [10] and stop-codon reassignment [3, 4]. Moreover, recent studies showed that *Euplotes* exhibits frequent PRF at stop codons [5, 6]. The frequent occurrence of ribosomal frameshifting during translation makes *Euplotes* an outstanding model for studying this universal phenomenon.

Using high-throughput sequencing approaches, we recently sequenced the macronuclear genome and the transcriptome of the typical species *E. octocarinatus*. We developed the *E. octocarinatus* Genome Database (EOGD) to provide the scientific research community with easy access to the genomic resources and information of *Euplotes*. EOGD provides user-friendly functions to access *Euplotes* genomic and transcriptomic data, as well as introduces the biology, taxonomy and morphology of *E. octocarinatus*. EOGD will serve as an important platform for researchers to facilitate research on *Euplotes* or other ciliates.

## Construction and content

EOGD integrates three major data sets of *E. octocarinatus*: (i) the macronuclear genome sequence data, (ii) the annotations of predicted genes, and (iii) the RNA-seq data of growth stage. Furthermore, EOGD provides a detailed description of the biology, taxonomy, and morphology of *E. octocarinatus*.

The *E. octocarinatus* macronuclear genome was assembled using a specialized meta-assembly pipeline based on Illumina sequences [5]. A total of 41,980 contigs with an average length of 2117 bp were obtained, and over 70% of these contigs were capped with telomeres on both ends [5]. Given that the existence of PRF genes will influence the accuracy of gene prediction, contigs known to contain frameshifted genes [5] were excluded from ab initio gene finding. AUGUSTUS, a *de novo* prediction software [11], was used to predict gene models on non-PRF contigs (38,615 in total). Of the 29,076 putative protein-coding genes that were obtained, 90% were supported by RNA-Seq reads [5].

The functional annotation of genomes, an important part of EOGD, is important in genomic studies. To obtain integrated functional genomic information, multiple annotations were performed. First, all putative proteins were functionally annotated with BLAST (Basic Local Alignment Search Tool) alignments against the NCBI non-redundant (nr) protein database. Second, conserved motifs and functional domains were predicted by InterProScan5 software [12] against the InterPro database [13]. The InterPro database provides comprehensive functional information on proteins from some partner databases, such as Pfam, PRINTS, PANTHER, Gene3D and InterPro. Gene Ontology (GO) annotations were performed by mapping of GO terms to Pfam entries [5]. This mapping was generated from data supplied by InterPro for the InterPro2GO [14] mapping. Finally, the clusters of orthologous groups across multiple ciliates, including *Tetrahymena thermophile*, *Paramecium tetraurelia*, *Ichthyophthirius multifiliis*, *Oxytricha trifallax*, and *Stylonychia lemnae*, were constructed by OrthoMCL [15].

Transcriptomic data are powerful resources for characterizing and validating gene models. EOGD stores the deeply sequenced RNA-seq data of *E. octocarinatus* growth stage. In brief, the *E. octocarinatus* transcriptome was sequenced with the Illumina deep RNA sequencing strategy. We obtained 39,478,354 short reads with a total length of more than 4.9 Gb [5]. Then the high-quality reads of RNA-seq data were mapped to the *Euplotes* macronuclear genome by Tophat [16]. The Bio::DB::SAM adaptor allows the Generic Genome Browser (GBrowse) to display the binary file as xyplot plots.

**Fig. 1:**
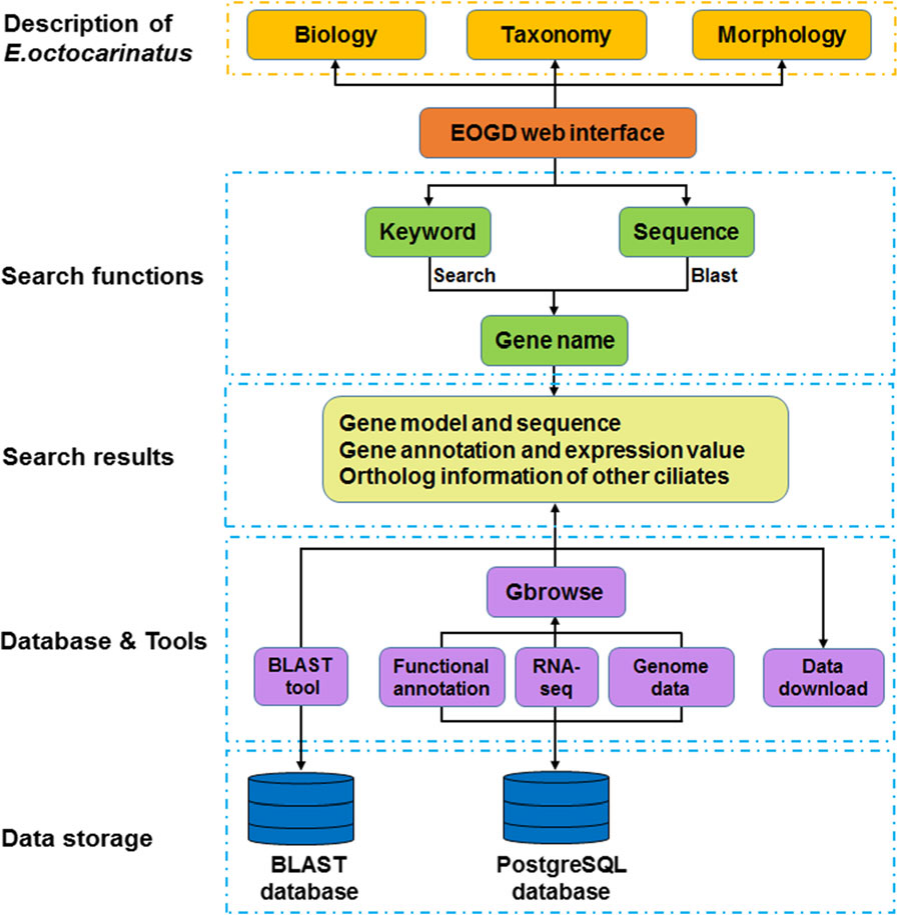
Schematic structure of EOGD. A flow diagram describing the database architecture is shown. Coding sequence, genome assembly data and protein sequence were formatted as BLAST database. Functional annotation information, RNA-seq data and genome data were stored in the PostqreSQL database. Keywords search and sequence search by BLAST allowed user to access the resources in EOGD easily. The EOGD also provides a description of *E. octocarinatus*

The schematic structure of EOGD is shown in Fig. 1. EOGD is built under the Linux system using a series of software packages, including Apache, PostgreSQL and Python. Genomic data and RNA-seq data were stored in the PostgreSQL database. The macronuclear genome sequences and gene sequences were formatted as a BLAST database. And we used Gbrowser [17], a widely used genome browser, to manipulate and visualize the genome annotations.

## Utility and discussion

**Fig. 2:**
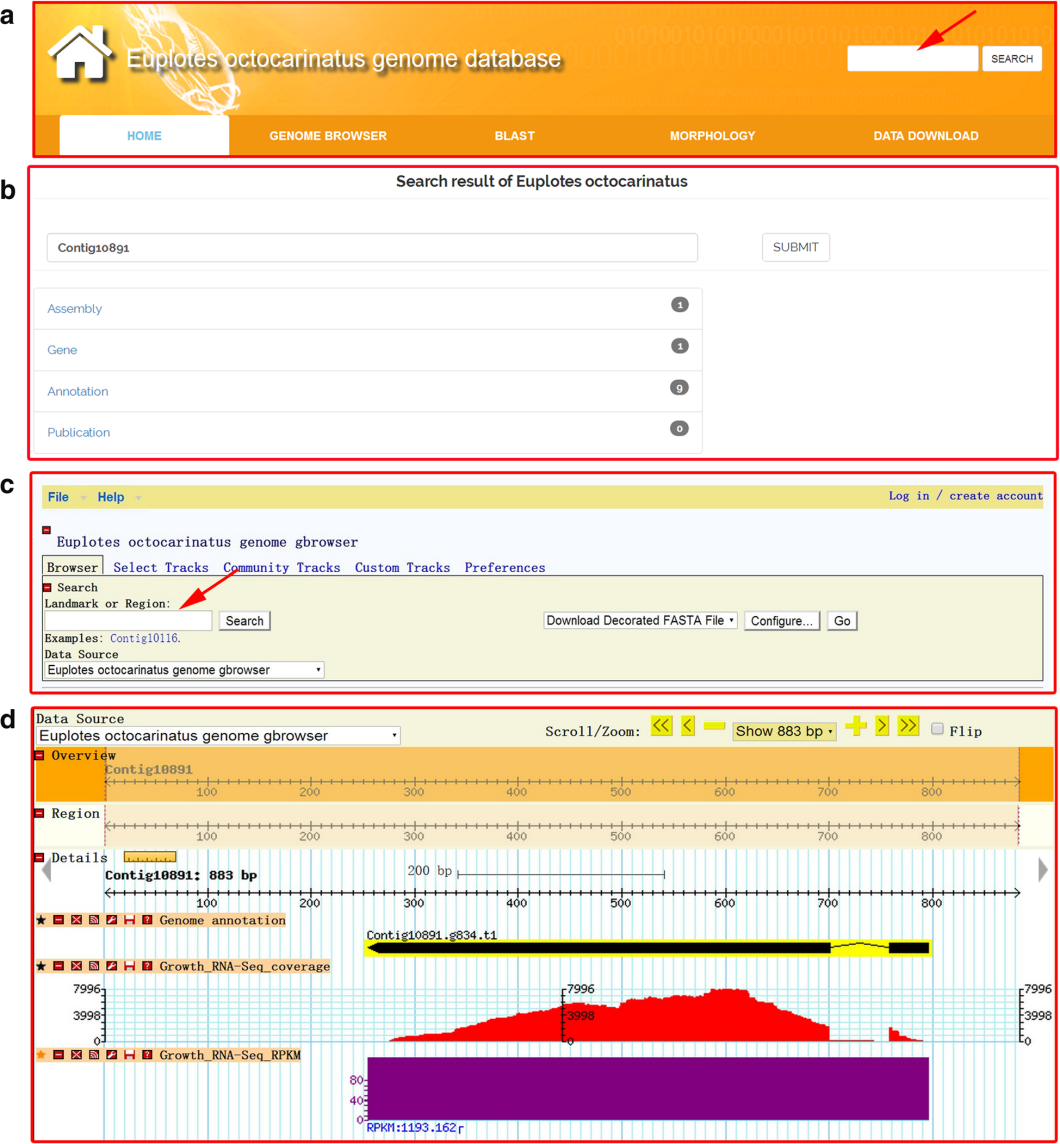
Web interface of EOGD. **a** Integrated searching box. The red arrow indicates the searching menu for selecting the “Gene ID”, “Genome ID” or “Keyword”. **b** Screenshot of search result interface of the EOGD website. (**c**–**d**) Gbrowse snapshots showing the search function interface (**c**) and search result interface (**d**) for Contig10891

EOGD can be accessed through a user-friendly web interface. The EOGD website contains navigation tabs (Home, Genome browser, Blast, Morphology and Data download) and a search box on top of each page (Fig. 2a).

EOGD provided two methods for users to rapidly search the genes of interest in the database: keywords and sequence search. If the user uses a “Keywords” to search, then any words (case insensitive) can be typed in the search box as search content. Scaffold ID and gene ID were also allowed to search specific genes. All searches will link to the result page (Fig. 2b). Furthermore, the user can search by a sequence. We implemented a server and an interface at our website for BLAST [18] search, which allows the user to search a query sequence against relevant *Euplotes* datasets (protein database, coding sequence database and genome database). Using BLAST tools, the user can obtain the detailed information of the sequence alignment for a region and download the BLAST results through the “DOWNLOAD” button.

EOGD graphically displays RNA-Seq and genome data and implements its search function through Gbrowse. Typically, the user can use “Gene ID”, “Scaffold ID” or a scaffold region to search the database (Fig. 2c). Three tracks, including a putative gene model track (linked to the gene sequence, annotation, and ortholog information), a RNA-Seq coverage plot track and a RPKM value track, are shown on the Gbrowse search result page (Fig. 2d). Through these tracks, the gene structure information and gene sequence can be obtained, which are important for exploring downstream biological functions. Any region of interest can be selected and the sequence can be downloaded in FASTA format by clicking the “Download Decorated FASTA File” in the pull-down menu or the “Export as FASTA sequence file” in the pull-down menu of the “File” button. Furthermore, the high-resolution image can be exported through Gbrowse.

**Fig. 3:**
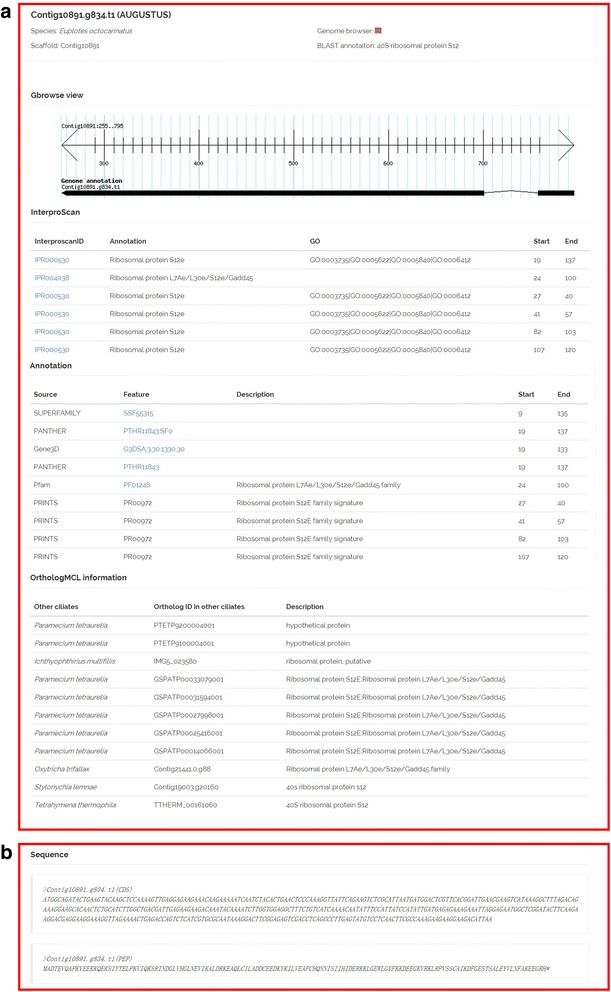
Case to illustrate the detailed search results. By clicking the predicted gene model on the Gbrowse search result page or searching the ID in the top search box, users can access the gene details page (taking the gene Contig10891.g834.t1 as an example). This gene details page will display all information for the gene of interest, including sequence name, scaffold ID, BLAST annotation, InterProScan domain information, annotated GO information, putative function annotation and ortholog information (**a**). Sequence information, including the nucleotide and amino acid sequence in FASTA format, will also be displayed (**b**)

By clicking the predicted gene model, users can obtain detailed information on the predicted gene, including annotations and sequence information (Fig. 3). Protein-coding gene models were annotated using BLAST alignments against the NCBI non-redundant (nr) protein database (E-value≤1e^− 5^). All predicted proteins were then functionally annotated (Fig. 3a). Functional domains and sites in all predicted protein models were identified by InterproScan (version Interproscan 5.2–45.0, run with default parameters) [12], which has combined signatures from a number of different database into one resource. All the predicted proteins were also annotated with GO for additional functional interpretation. In addition, OrthoMCL [15] was used to construct orthologous groups across multiple ciliates. The “Sequence” section includes the nucleotide and amino acid sequence of the predicted gene (fasta format) (Fig. 3b).

On the “MORPHOLOGY” page (http://ciliates.ihb.ac.cn/database/morphology/#eo), the microscopic morphology and the detailed taxonomic classification of *E. octocarinatus* are described according to several previous studies [19–21]. Furthermore, EOGD provides a data download page (http://ciliates.ihb.ac.cn/database/download/#eo) for downloading *E. octocarinatus* genome sequences, protein sequences, coding sequences in FASTA format and annotation information. User can download these data by clicking the “download” button.

### Further development of the EOGD

EOGD will be periodically updated. Additional *Euplotes* data sets, such as micronuclear genome, mitochondrial genome and proteome data, as well as gene expression profiles, will be uploaded to and integrated in EOGD in the future. Given the high-throughput and relatively low cost of next-generation sequencing, sequencing the genomes and transcriptomes of other *Euplotes* species in the future is possible.

## Conclusions

EOGD allows researchers to access, browse, retrieve and analyze genomic and transcriptomic data and annotations. This hub will promote research on *Euplotes* biology, and will also enhance our understanding of eukaryotic molecular biology. We will periodically update EOGD by integrating more data. EOGD is an important and useful resource that is freely available to the research community.

## Abbreviations

BLAST: Basic Local Alignment Search Tool
EOGD: *Euplotes octocarinatus* Genome Database
GO: Gene ontology
MAC: macronucleus
MIC: micronucleus
NCBI: National Center for Biotechnology Information
RPKM: Reads Per Kilobase Per Million Mapped Reads
